# The inactivation of RNase G reduces the *Stenotrophomonas maltophilia* susceptibility to quinolones by triggering the heat shock response

**DOI:** 10.3389/fmicb.2015.01068

**Published:** 2015-10-19

**Authors:** Alejandra Bernardini, Fernando Corona, Ricardo Dias, Maria B. Sánchez, Jose L. Martínez

**Affiliations:** ^1^Departamento de Biotecnología Microbiana, Centro Nacional de Biotecnología, Consejo Superior de Investigaciones CientíficasMadrid, Spain; ^2^Biosystems and Integrative Sciences Institute, Faculty of Sciences, University of LisbonLisbon, Portugal

**Keywords:** *Stenotrophomonas maltophilia*, quinolone resistance, antibiotic resistance, RNase G, heat shock

## Abstract

Quinolone resistance is usually due to mutations in the genes encoding bacterial topoisomerases. However, different reports have shown that neither clinical quinolone resistant isolates nor *in vitro* obtained *Stenotrophomonas maltophilia* mutants present mutations in such genes. The mechanisms so far described consist on eﬄux pumps’ overexpression. Our objective is to get information on novel mechanisms of *S. maltophilia* quinolone resistance. For this purpose, a transposon-insertion mutant library was obtained in *S. maltophilia* D457. One mutant presenting reduced susceptibility to nalidixic acid was selected. Inverse PCR showed that the inactivated gene encodes RNase G. Complementation of the mutant with wild-type RNase G allele restored the susceptibility to quinolones. Transcriptomic and real-time RT-PCR analyses showed that several genes encoding heat-shock response proteins were expressed at higher levels in the RNase defective mutant than in the wild-type strain. In agreement with this situation, heat-shock reduces the *S. maltophilia* susceptibility to quinolone. We can then conclude that the inactivation of the RNase G reduces the susceptibility of *S. maltophilia* to quinolones, most likely by regulating the expression of heat-shock response genes. Heat-shock induces a transient phenotype of quinolone resistance in *S. maltophilia*.

## Introduction

*Stenotrophomonas maltophilia* is a nosocomial opportunistic pathogen that is considered a prototype of intrinsically resistant bacterium. (Brooke, 2012) The characteristic low-susceptibility of this organism to different antibiotics mainly relies in the presence in its genome of genes encoding several intrinsic resistance elements that include antibiotic-inactivating enzymes, multidrug eﬄux pumps and a quinolone resistance protein; SmQnr. (Walsh et al., 1997; Lambert et al., 1999; Alonso and Martinez, 2000; Avison et al., 2002; Okazaki and Avison, 2007; Crossman et al., 2008; Sanchez et al., 2008; Shimizu et al., 2008; Al-Hamad et al., 2009; Sanchez and Martinez, 2010; Garcia-Leon et al., 2014b) Quinolones are synthetic antimicrobials which targets are the bacterial topoisomerases. Given their synthetic origin, it was expected that quinolone resistance genes should be absent in natural ecosystems and the only mechanism of resistance would be mutations in the genes encoding bacterial topoisomerases. Further work demonstrated this not to be true; overexpression of eﬄux pumps can confer quinolone resistance, and the acquisition of genes encoding target-protecting proteins (quinolone resistance protein, Qnr) renders resistance to these antimicrobials as well (Courvalin, 1990; Hernandez et al., 2011).

Despite these findings, mutations at the genes encoding topoisomerases still remain as the most important mechanism conferring high-level quinolone resistance in clinically relevant bacteria. The only exception is *S. maltophilia*. Different studies have shown that neither clinical isolates presenting high-level quinolone resistance nor *in vitro* selected quinolone resistant mutants present mutations in genes encoding *S. maltophilia* topoisomerases (Ribera et al., 2002; Valdezate et al., 2002; Garcia-Leon et al., 2014b). The best studied mechanisms of quinolone resistance in this bacterial species consist on the overexpression of the multidrug eﬄux pumps SmeDEF or SmeVWX (Alonso and Martinez, 2001; Gould and Avison, 2006; Garcia-Leon et al., 2014b). Nevertheless, some quinolone resistant clinical isolates neither overproduce any of the already described *S. maltophilia* multidrug eﬄux pumps not present mutations on the genes encoding bacterial topoisomerases (Garcia-Leon et al., 2014b, 2015). This indicates that there are still mechanisms of quinolone resistance, including overexpression of other eﬄux pumps as SmrA, which is known to extrude quinolones (Al-Hamad et al., 2009) that remain to be unveiled in *S. maltophilia*. Consequently with this situation, we decided to screen a transposon-tagged insertion library in the aim of finding novel mechanisms of quinolone resistance in *S. maltophilia*. A recent article has reviewed the different definitions of resistance: based on clinically relevant breakpoints, on ecologically relevant breakpoints or in the comparison of two isogenic strains, the resistant one presenting a higher MIC than its parental (Martinez et al., 2015). Along this work we will make use of this third definition of resistance, which is the best suited for determining novel mechanisms of resistance irrespectively, on the level of MIC increase achieved. (Martinez et al., 2015) Using this criterion, we found that the inactivation of RNase G increases the quinolone MICs of *S. maltophilia*. The inactivation of RNases has been associated in few cases with changes in the susceptibility to antibiotics; however, in most published articles and opposite to our findings, such inactivation increases the susceptibility to the analyzed antimicrobials (Saramago et al., 2014).

The role of RNase G on *S. maltophilia* should be indirect by regulating the level of expression of the genes actually conferring resistance. Because of this, we analyzed the effect of inactivating this enzyme on *S. maltophilia* transcriptome and tracked the cause of resistance to the heat-shock response. It has been shown that antibiotics can trigger different stress responses and that such responses can in occasions produce a transient reduction in the susceptibility to antibiotics of bacterial pathogens (Utaida et al., 2003; Cardoso et al., 2010; Kindrachuk et al., 2011). Our data are consistent with these findings and support that mutations producing a de-repressed constitutive expression of the heat-shock response can reduce the susceptibility of *S. maltophilia* to quinolones.

## Materials and Methods

### Bacterial Strains and Growth Conditions

The bacterial strains used were the *S. maltophilia* clinical strain D457 (Alonso and Martinez, 1997), the *S. maltophilia* D457 insertion mutant ALB001 and their isogenic derivatives ALB002 [D457(pVLT33)], ALB003 [D457(pVLT33-*rng*)], ALB004 [ALB001(pVLT33)], and ALB005 [ALB001(pVLT33-*rng*)]. *Escherichia coli* CC118aaapir, S17-1 with pUT-miniTn5::Tc and *E. coli* 1047/pRK2013 strains were used for conjugation (de Lorenzo and Timmis, 1994). All strains were grown in LB medium at 37°C, unless indicated.

### Generation of Transposon Insertion Mutant Libraries of *S. maltophilia* D457

Random transposon insertion mutant libraries were generated in the *S. maltophilia* D457 strain (Alonso and Martinez, 1997) as described using the minitransposon mini-Tn*5*-*Tc* (de Lorenzo et al., 1990). The transposon was transferred to *S. maltophilia* D457 by conjugation (de Lorenzo and Timmis, 1994) using the donor strain *E. coli* S17-1 aaa*pir* (pUT mini-Tn*5*-*Tc*) (de Lorenzo et al., 1990) and the helper strain *E. coli* 1047/pRK2013 (Figurski and Helinski, 1979). One ml aliquots of overnight cultures of each strain were centrifuged 3 min at 5900x *g*. The pellets were suspended in 1 ml of 10 mM MgSO_4_. For the mating, suspensions were mixed at a 1:10:10 (receptor:donor:helper) ratio, incubated for 10 min at 42°C and then 5 min at 4°C. Cell mixtures were filtered through 0.45 μm filters (Millipore). The filters were placed onto LB agar (LBA) plates and incubated at 30°C during 8 h.

Cells grown on the filter surface were suspended in 5 ml of M9 medium and 200 μl of each suspension were spread in LBA plates containing 10 mg/L tetracycline and 20 mg/L imipenem. The number of colonies was recorded after 24 h of incubation. The colonies were recovered in 1 ml of M9 medium, centrifuged and recovered in PBS with 20% glycerol to store at -80°C.

### Antibiotic Susceptibility Assays

The MICs of the different antibiotics were determined on Mueller Hinton agar plates by MIC Test Strip (Liofilchem). The antibiotics used were ciprofloxacin, gatifloxacin, levofloxacin, norfloxacin, nalidixic acid, erythromycin, tigecycline, cotrimoxazole, and ceftazidime. The results were recorded after 24 h of incubation at 37°C.

### Inverse-PCR

Inverse-PCR was performed, mainly as described (Fajardo et al., 2008), to identify the gene interrupted by the transposon in the mutant strain ALB001. Chromosomal DNA was obtained using the GNOME DNA kit (MP Biomedicals). One micro gram of genomic DNA was cut using 20 units of the restriction enzyme PstI (Biolabs Inc.) at 37°C for 2 h. One hundred ng of the digested DNA were auto-ligated using 200 units of T4 DNA ligase (BioLabs Inc.) at 16°C overnight in 200 μl of ligation buffer (50 mM Tris-HCl, 10 mM MgCl_2_, 10 mM dithiothreitol, 1 mM ATP, 25 mg/ml bovine serum albumin, pH 7.5). After ligation, DNA was precipitated by adding 20 μl of 3 M sodium acetate, pH 5.2 and 500 μl of ethanol 100%. The mixture was incubated 20 min at 4°C, and the DNA was pelleted by centrifugation. DNA was washed with ethanol 70%, dried at 37°C, resuspended in water and used for inverse-PCR of the circularized fragments using the primers Tn5A2 and Tn5A3 (**Table 1**). The PCR reaction was made in 50 μl reaction mixture containing 5 μl of PCR buffer 1 10X from the Expand Long Template PCR System (Roche), each deoxynucleotide at a concentration of 350 μM, 300 nM of each primer, 2.5 units of Expand Long Template Enzyme mix (Roche) and the template DNA obtained as described above. The mixture was heated at 94°C for 2 min, followed by 30 cycles of 10 s at 94°C, 30 s at 55°C and 6 min at 68°C, and finally one 7 min extension step at 68°C. The PCR product was run on 1% agarose gel (Sambrook and Russell, 2001) and purified using the QIAquick PCR purification kit (Qiagen). The amplicon was sequenced with the same primers used for the PCR in the Parque Científico of Madrid.

**Table 1:** Primers used along this work for DNA amplification.

Primers	5′-3′ sequence	Description
Tn5 A3	gccttgatgttacccgagagc	Used for inverse-PCR
Tn5 A2	aaaatctagcgagggctttac	Used for inverse-PCR
RNaseF	cggaattcccgatgtctgaggaa	To amplify and clone *rng* (*Eco*RI target underlined)
RNaseR	cccaagcttggatcagagcagaa	To amplify and clone *rng* (*Hin*dIII target underlined)
Sme27	tgccagcgacagtgcaaagggtc	To amplify a *Stenotrophomonas maltophilia* specific region (Sanchez et al., 2002)
Sme48	ccgtgttcatggaagcaggc	To amplify a *S. maltophilia* specific region (Sanchez et al., 2002)

### Complementation of *S. maltophilia* ALB001 Strain with the *rng* gene of *S. maltophilia* D457

The *rng* gene of *S. maltophilia* D457 was amplified using the primers RNaseF and RNaseR (**Table 1**). The reaction was performed using the Expand Long Template PCR system (Roche), 500 ng of genomic DNA of *S. maltophilia* D457 as template, 350 μM of each deoxynucleotide, and 0.5 μM of each primer. The reaction had one denaturation step at 94°C for 5 min, followed by 30 amplification cycles of 94°C for 30 s, 55°C for 30 s, and 68°C for 1.5 min, with a final extension step of 68°C for 7 min. The PCR product was cloned into the pGEM-T plasmid (Promega), generating the pGEM-T-*rng* plasmid. The inserted fragment was sequenced by Macrogen^1^ to ensure that no mutations were introduced during PCR. The pGEM-T-*rng* plasmid was *Eco*RI-*Hin*dIII digested, and the fragment containing the *rng* gene was purified and cloned into pVLT33 (de Lorenzo et al., 1993), in the same enzyme restriction sites. The plasmid containing the wild-type allele of *rng* was dubbed pBA01. This plasmid as well as the cloning vector pVLT33 were introduced into *E. coli* CC118aaapir and mobilized into *S. maltophilia* D457 and ALB001, using the *E. coli* 1047/pRK2013 strain as a helper, by triple conjugation (de Lorenzo and Timmis, 1994) at a rate of 4:1:2 (receptor-donor-helper) and using M9 (Atlas, 1993) to recover the mating mixture from the filter. The exconjugants were selected on LB plus 300 mg/L kanamycin and 20 mg/L imipenem and the presence of the pVLT33 and pBA01 plasmid was checked by extracting the plasmid by QIAprep Spin Miniprep Kit (Qiagen) and pBA01 was further analyzed by *Eco*RI-*Hin*dIII digestion and electrophoresis on a 0.8% agarose-ethidium bromide gel.

### RNA Preparation and Real-time RT-PCR

Flasks containing 25 ml LB were inoculated with either *S. maltophilia* D457 or ALB001 overnight cultures to 0.01 OD_600nm_ and were grown at 37°C until an OD_600nm_ of 0.6 was reached; in order to synchronize growing cells, new flasks containing 25 ml LB were inoculated with the aforementioned cultures to 0.01 OD_600nm_ and grown at 37°C until an OD_600nm_ of 0.6 was reached. Ten milliliters of cultures were mixed with 10 ml of RNAprotect Bacteria Reagent (Qiagen) during 10 min at room temperature. Afterward, cells were spun down at 6,000× g for 10 min at 4°C and immediately frozen on dry ice and stored at -80°C. Total RNA extraction, DNA elimination, RNA integrity verification, DNA absence confirmation, cDNA generation, and real-time PCR were performed as described previously (Olivares et al., 2012). Total RNA was extracted from cell pellets by RNeasy mini- kit (Qiagen) according to the manufacturer’s instructions. To further eliminate any remaining DNA, Turbo DNA-free (Ambion) was used. RNA integrity was verified on a 1% agarose gel, and the absence of DNA was verified by PCR using primers Sme27 and Sme48 (**Table 1**). cDNA was obtained from 5 μg RNA by a High Capacity cDNA reverse transcription (RT) kit (AB Applied Biosystems). Real time PCR mixture was obtained using the Power SYBR green kit (Applied Biosystems) as indicated by the manufacturer and the reaction was performed as follows; a first denaturation step, 95°C for 10 min, was followed by 40 temperature cycles (95°C for 15 s, 60°C for 1 min). Differences in the relative amounts of mRNA for the different genes were determined according to the 2^-ΔΔ^*^CT^* method (Livak and Schmittgen, 2001). In all cases, the mean values for relative mRNA expression obtained in three independent experiments, each one with three technical replicates were considered. Expression of the reference gene *rpoD* was used for the normalization of the results (Garcia-Leon et al., 2014a). The primers used for the real-time PCR are indicated in **Table 2**.

**Table 2:** Primers used along this work for real time RT-PCR.

Primers	5′-3′ sequence	Final concentration (nM)
dnaK 1	gcgtcatcgagtacctggtt	400
dnaK 2	caggttcacttcggtctgct	400
emrA 1	atgagccagacccaagacac	200
emrA 2	ggccgaacatgaagtaccac	200
emrB 1	agcacatctcggcctatcag	200
emrB 2	gatgtcgttgaagcccatct	200
groEL 1	aagaaggtgcaggtctccaa	200
groEL 2	tcgtaatccgaggaggtgtc	200
groES 1	ccaaggaaaagtccaccaag	200
groES 2	cgtactggccgtagatgacc	200
grpE 1	caagttcgccaacgagaag	200
grpE 2	tgcttgtaggtcagctccag	200
hslU 1	agaccgaccacatcctgttc	600
hslU 2	cacgaaatcgttcttgctca	800
htpG 1	catcaccatcgaagacaacg	600
htpG 2	agctgcgaatccttcttctg	800
clpB 3	acttcaagctggtgcaggac	400
clpB 4	gttgtgcagcacttcttcca	400
dnaJ 3	aagcctacgaagtgctgtcc	400
dnaJ 4	ccaaaaatgttgccgaagat	400
hslV 3	ggtggctcgtatgcactgtc	600
hslV 4	acgttgcggttggtgtagat	800
rnasaG 1	gaggacatcgcctacctgtc	200
rnasaG 2	accttcaccttgtccacgtc	200
rpoD1	ggtgcacatgatcgaaacga	500
rpoD2	gccgtactgctggagcatct	500

### Transcriptomic Analysis

To assess the transcriptome of *S. maltophilia* D457 and ALB001 strains, RNA was obtained from three independent cultures of each strain. The triplicates RNAs from each strain were then pooled to reduce biological variability. After elimination of rRNA with RiboZero, cDNAs were synthesized and sequenced using the Illumina technology in a 75 bp-single-end format at the Parque Científico of Madrid. The data of RNA-sequencing were normalized as reads per kilobase per million mapped reads (RPKM; Mortazavi et al., 2008) analyzed, and visualized using Fiesta 1.1^2^.

### Determination of mRNAs Half Lives

A rifampicin run-out experiment was conducted by adding rifampicin 200 mg/L to synchronized cultures (OD_600nm_ 0.6) and taking samples every 5 min after transcription inhibition by rifampicin. RNA extraction and real-time RT-PCR were carried out as described above. The values of half-lives were estimated from the mean decay rate for each mRNA.

### Killing Curves under Heat Shock

The killing curves of *S. maltophilia* D457 and ALB001 with or without plasmid pBA01 were established, under antibiotic pressure and at different temperatures. The different strains were inoculated from overnight cultures and grown at 37°C until they reached an OD_600nm_ of 0.6. Then, cultures were diluted 1:10 on LB medium containing nalidixic acid 48 mg/L and grown either at 37°C or at 42°C for 60 min. Ten minutes after heat shock, RNA was extracted from the different cultures and used for real-time RT-PCR as described above. At different times after inoculation, samples were taken and serial dilutions of such samples were plated in LBA Petri dishes. Colony-forming-units (cfu) were recorded after 24 h of incubation at 37°C.

## Results and Discussion

### Generation of a *S. maltophilia* Transposon-insertion Library and Selection of a Quinolone Resistant Mutant

Four independent conjugations were performed as described in methods to obtain different libraries. Each library contained between 1600 and 2000 mutants. The whole library then contains around 7000 independent mutants. A screening was performed by plating the library on Mueller–Hinton agar Petri dishes containing nalidixic acid 128 mg/L. Selected mutants were grown in medium without antibiotic (two passages) to assure that the observed phenotype was not transient and the susceptibility to quinolones was tested by disk diffusion (not shown). One mutant presenting a decreased susceptibility to different quinolones was chosen for further studies and dubbed ALB001.

To determine the gene in which the transposon had been inserted, an inverse PCR reaction was performed as described in methods and the amplified DNA was sequenced using Sanger technology. Comparison of the sequence with the *S. maltophilia* D457 genome (Lira et al., 2012) showed that the transposon was inserted inside the SMD_3054 gene, which encodes an ortholog of RNase G. To further confirm the position of the transposon, a PCR was performed using primers flanking the gene encoding RNase G. Confirming the results of the inverse PCR, the size of the amplicon from ALB001 fits with that predicted for the presence of the miniTn5 inside *rng* (not shown).

To further confirm the role of *rng* inactivation in the observed phenotype, the wild-type allele of *rng* was cloned and expressed in the wild-type *S. maltophilia* strain D457 and the *rng* defective mutant ALB001 as described in Methods. The strains were also transformed with the pVLT33 vector and the resultant strains used as controls. The susceptibility of the different strains to different quinolones was then analyzed by using MIC Test Strip. As shown in **Table 3**, the *in-trans* expression of the wild-type allele of *rng* in ALB001 reduced the susceptibility of the mutant to levels close to those of the wild-type strain. To address whether or not the inactivation of *rng* also alters *S. maltophilia* susceptibility to other non-quinolone antibiotics, the MICs of erythromycin, tigecyclin, cotrimoxazole, and ceftazidime were measured. We found that the inactivation of *rng* did not produce any effect on *S. maltophilia* susceptibility to these non-quinolone antimicrobials. Altogether, these results further support that the inactivation of RNase G reduces the susceptibility to quinolones of *S. maltophilia*.

**Table 3:** Susceptibility to quinolones of the strains used in this work.

Strains	MIC (mg/L)								
	Nalidixic acid	Norfloxacin	Levofloxacin	Ciprofloxacin	Gatifloxacin	Erythromycin	Tigecycline	Cotrimoxazole	Ceftazidime
D457	6	12	0.5	1.5	0.5	128	1	0.125	2
D457 (pVLT33)	4	12	0.5	1.5	0.38	128	1.5	0.125	1.5
D457 (pBA001)	4	12	0.5	1.5	0.38	128	1	0.125	1.5
ALB001	64	32	1.5	3	0.75	128	1.5	0.125	1.5
ALB001 (pVLT33)	128	48	3	6	1	128	1.5	0.125	1.5
ALB001 (pBA001)	8	12	0.75	2	0.5	128	1.5	0.094	1.5

### The Inactivation of RNase G alters the Transcriptome of *S. maltophilia*

**FIGURE 1:**
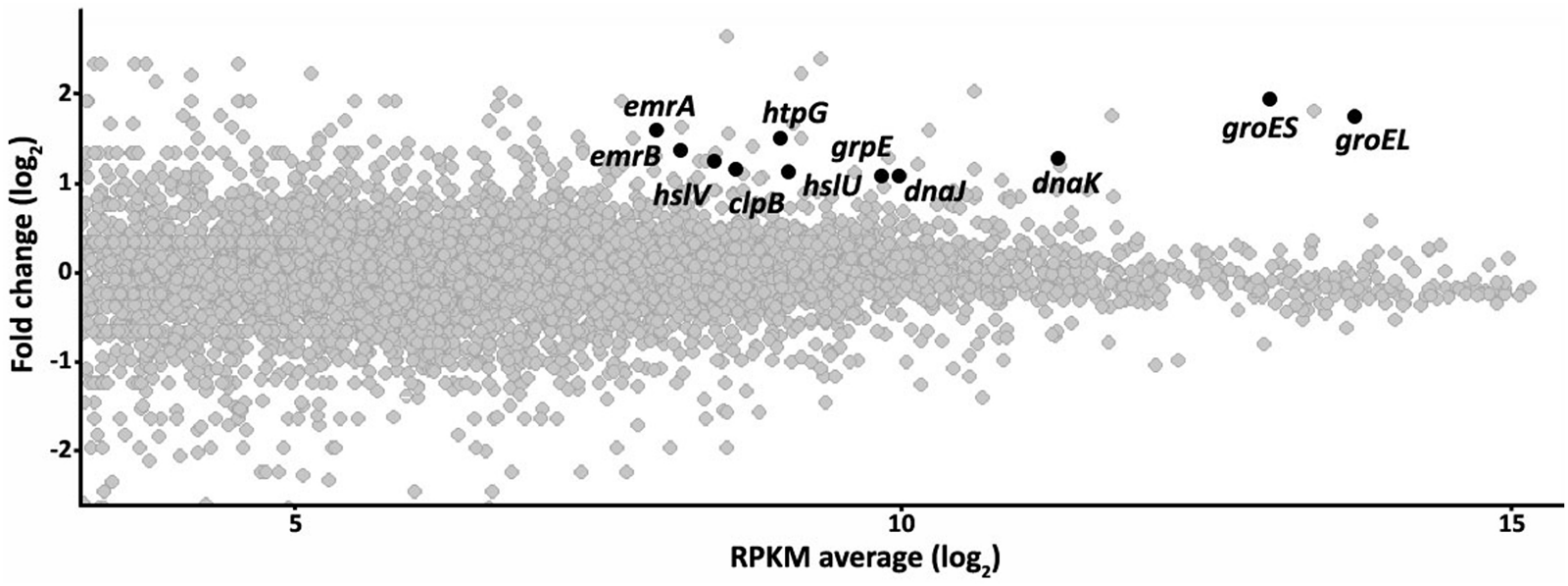
**The inactivation of the RNase G alters the transcriptome of *Stenotrophomonas maltophilia*.** The effect of inactivating the RNase G in the transcriptome of *S. maltophilia* was studied by RNA sequencing as described in the text. The figure represents the fold change in the expression level of RNAs from the RNase G-defective mutant ALB001 in comparison with the wild-type strain D457, as a function of the average of the RPKM values gene in both strains. The Figure is represented in a log_2_ scale. Genes, presenting higher expression in the RNase G defective mutant and selected for further studies are highlighted.

Since RNases are involved in RNA processing and the targets of the quinolones are the bacterial topoisomerases, a direct effect of the inactivation of RNase G on quinolone susceptibility is unlikely. Indeed, it has been reported that in *E. coli* RNase G is involved in the regulation of the central metabolism (Lee et al., 2002; Sakai et al., 2007). The observed resistance might be due to another determinant(s) the expression of which is regulated by RNase G. To have further insights of these putative elements, the transcriptome of the ALB001 RNase G defective mutant was analyzed by Illumina sequencing in comparison with that of the wild-type strain D457. As shown in **Figure 1** and Supplementary Table S1, several genes were expressed at a higher level in the mutant ALB001 than in the wild-type strain D457.

### The Inactivation of RNase G Increases the Expression of Genes Involved in *S. maltophilia* Heat Shock Response

Among the genes that were overexpressed in the ALB001 mutant when compared with the isogenic parental strain D457, some of them encoded proteins forming part of the heat shock response. The genes *emrA* and *emrB*, which encode a multidrug eﬄux pump were also expressed at a higher level in ALB001 than in D457. RpoH is an alternative sigma factor involved in the heat-shock response (Grossman et al., 1984). Our transcriptomic study shows that *rpoH* expression in slightly higher (1.4-fold change) in the *rng* mutant as compared with the wild-type strain. Whether or not this slight increase in the expression of this transcriptional activator is enough for triggering the heat-shock response remains to be elucidated.

**FIGURE 2:**
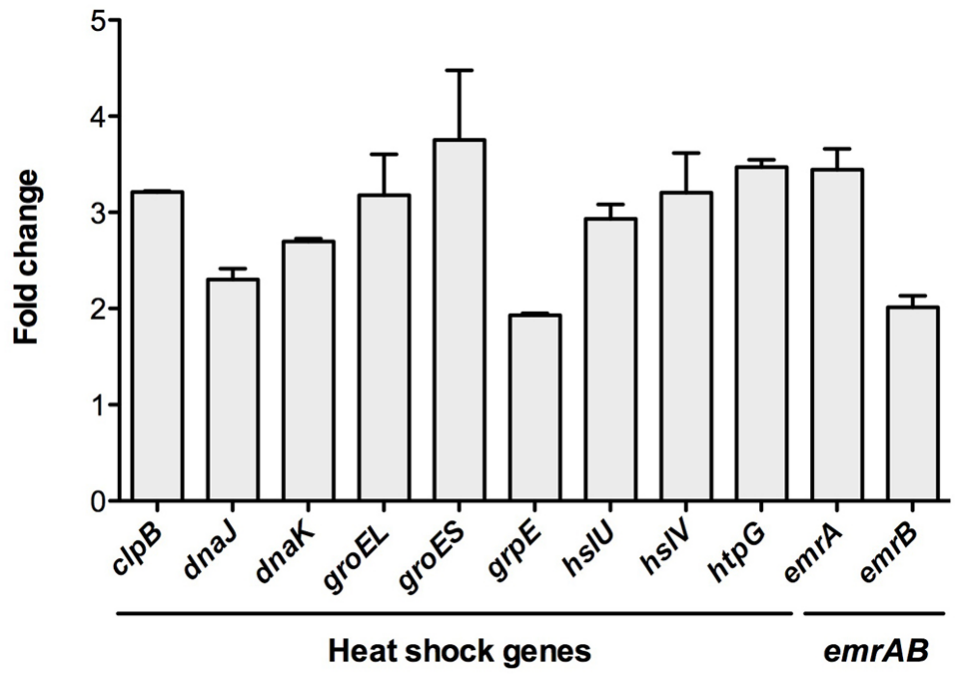
**The inactivation of RNase gene increase the expression of heat-shock response genes.** To confirm the transcriptomic assays, the expression of the genes *clpB, dnaJ, dnaK, groEL, groES, grpE, hslU, hslV, htpG, emrA*, and *emrB* was determined by real-time RT-PCR in the wild-type strain and the RNase defective mutant ALB001. As shown, all genes were expressed at a higher level in the RNase G defective mutant, confirming the results of the RNA-seq analysis.

To further confirm the transcriptomic studies, the expression of this set of selected genes was measured by real-time RT-PCR as described in Methods. As shown in **Figure 2**, for all the selected genes, namely *clpB, dnaJ, dnaK, groEL, groES, grpE, hslU, hslV, htpG, emrA*, and *emrB*, expression was higher in the RNase G defective mutant than in the wild-type strain. This result further confirms that RNase G down-regulates *emrA*, *emrB* and different genes encoding proteins involved in *S. maltophilia* heat-shock response. It would be possible that these genes are targets of RNase G, in which case the half lives or their messenger RNAs will be longer. For addressing this possibility a rifampicin run-out experiment was conducted and half-lives of *groEL* and *groES* mRNAs were calculated in the wild-type strain and in the RNase G defective mutant. As shown in **Table 4**, inactivation of *rng* led to a minor increase in the half lives of *groEL* and *groES* mRNAs (10 and 20%), suggesting these mRNAs are not direct targets of RNase G.

**Table 4:** Effect of RNase G on the stability of *groES* and *groEL* mRNAs.

Gene	mRNA half-life (min)		Increase (*%*)
	D457	ALB001	
*groES* (SMD_3814)	3,65	4,01	10
*groEL* (SMD_3813)	3,69	4,41	20

Overexpression of multidrug eﬄux pumps is involved in the acquisition of resistance to quinolones in *S. maltophilia* (Chang et al., 2004; Sanchez et al., 2004; Gould and Avison, 2006; Garcia-Leon et al., 2014b). Indeed, it has been shown that EmrAB overexpression protects *E. coli* from the antibiotics nalidixic acid and thiolactomycin in *E. coli* (Lomovskaya et al., 1995). It is then possible that the responsible for the resistance of the ALB001 mutant would be the overexpression of the EmrAB eﬄux pump. (Lomovskaya et al., 1995).

In addition, it has been shown in *E. coli* that the inhibition of the heat shock chaperon DnaK (Credito et al., 2009) as well as mutations in the heat-shock-response genes *dnaK, groEL*, and *lon*, increase the susceptibility to quinolones (Yamaguchi et al., 2003). If the heat-shock response is involved in the intrinsic resistance to quinolones, it might be speculated that triggering the heat-shock response might induce resistance. In this regard, it is worth mentioning that the inhibition of the DNA gyrase, the target of quinolones, induces the heat shock response in *E. coli* (Kaneko et al., 1996).

### The Heat-shock Response Reduces the Susceptibility to Quinolones of *S. maltophilia* without Increasing *emrA, emrB* Expression

**FIGURE 3:**
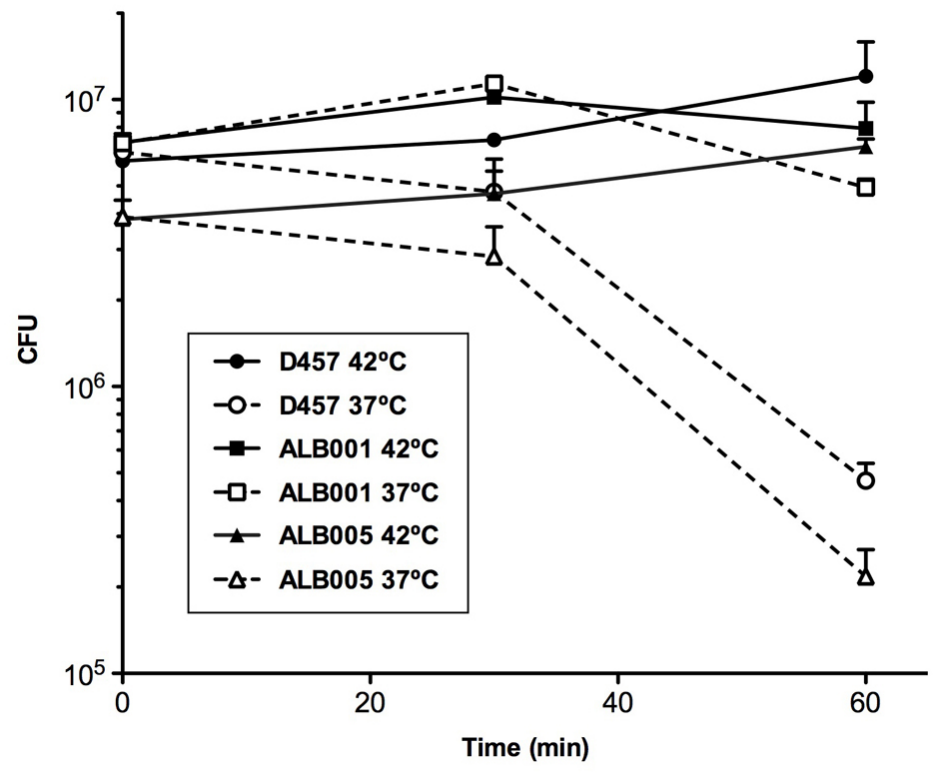
**Heat-shock reduces the susceptibility of *S. maltophilia* to nalidixic acid.** The wild-type strain *S. maltophilia* D457 and its isogenic, RNase G defective mutant ALB001, were either grown at 37°C or subjected to a 42°C heat-shock in the presence of 48 mg/L of nalidixic acid (NA) and the killing curves of each of the cultures were determined. As shown, the wild-type strain D457 was killed by the quinolone when growing at 37°C, but it was not affected under heat-shock conditions. The RNase defective mutant ALB001 was not affected by quinolones under any of the tested conditions. The means and standard deviations of three independent experiments are shown.

**FIGURE 4:**
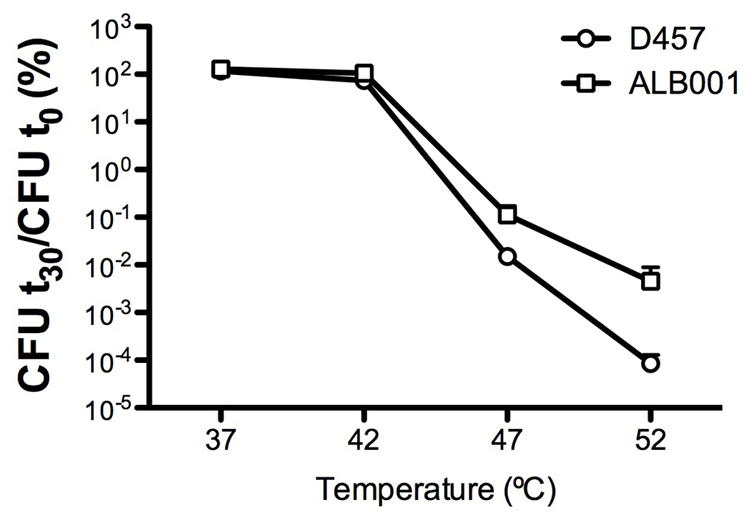
**The lack of RNase G increases the survival of *S. maltophilia* at high temperatures.** The wild-type strain *S. maltophilia* D457 and its isogenic, RNase G defective mutant ALB001, were grown at either 37, 42, 47, or 52°C. Colony forming units (CFUs) were counted after 30 min of incubation. As shown, the RNase defective mutant ALB001 survived better than the wild-type strain when growing at 47 and 52°C. The means and standard deviations of three independent experiments are shown.

To determine whether or not a heat-shock might reduce the susceptibility to quinolones of *S. maltophilia*, the kinetic of death in the presence of the quinolone nalidixic acid of the different strains was measured for bacteria growing at 37°C and upon a 42°C heat-shock (**Figure 3**). As shown, more than 90% of the population of the wild-type strain D457 was killed after 60 min of incubation at 37°C in the presence of nalidixic acid, whereas the quinolone did not exert any effect over the heat-shocked D457 population. Consistent with the role of the inactivation of RNase G on quinolone resistance, growth of the ALB001 mutant was inhibited neither at 37°C nor upon heat-shock. Notably, *in-trans* expression of the wild-type allele of *rng* restored the quinolones susceptibility of the ALB001 mutant to the levels of the wild-type strain D457 when growing at 37°C (**Figure 3**). Further supporting a role of RNase G on *S. maltophilia* heat shock response and its survival at high temperatures is the finding that the ALB001 mutant survives better than the wild-type strain upon incubation at high temperatures (47 or 52°C; **Figure 4**).

**FIGURE 5:**
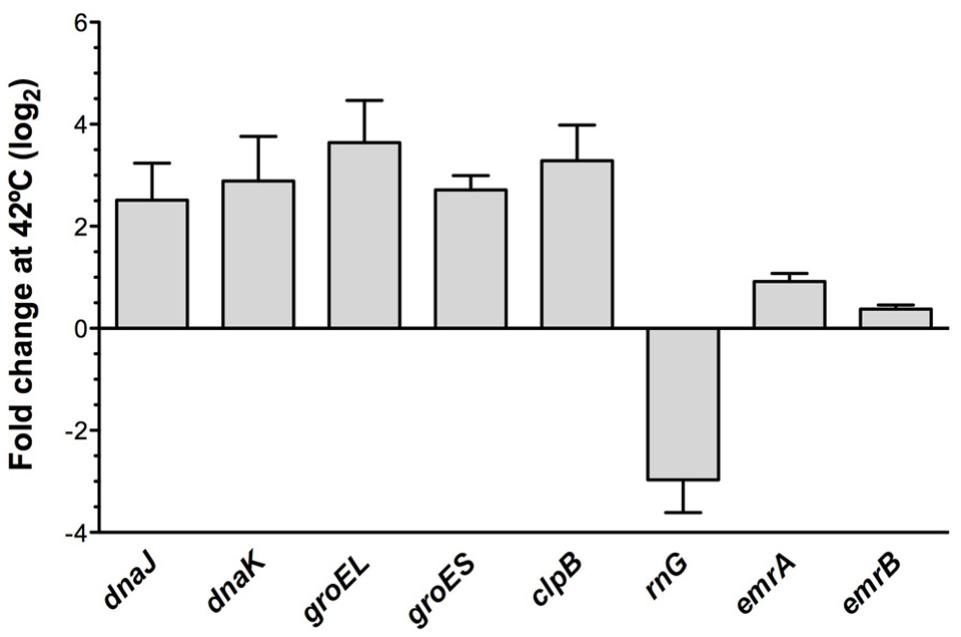
**Expression of heat-shock genes, *rng, emrA*, and *emrB* under heat shock conditions.** The wild-type strain *S. maltophilia* D457 was grown either at 37 or under heat-shock conditions at 42°C. RNA was isolated after 10 min of incubation and the expression of *clpB, dnaJ, dnaK, groEL, groES, rng, emrA*, and *emrB* was measured by real time RT-PCR. Gene expression at 42°C relative to 37°C. As shown, all the analyzed heat shock genes are over-expressed under heat shock conditions. While, opposite to this situation, *rng* expression is down-regulated after a heat shock. Notably, expression of *emrA* and *emrB* is not significantly affected by the heat shock.

Since RNase G downregulates expression of heat-shock genes at permissive temperature, it is possible that the induction of the expression of such genes under heat shock is due to the down-regulation of the expression of *rng* under such conditions. To address this possibility, the expression of the heat shock genes *dnaJ, dnaK, groEL, groES, clpB* as well as *rng, emrA*, and *emrB* was measured at 37°C and upon heat-shock conditions. As shown in **Figure 5**, *rng* is expressed at lower levels under the same heat-shock conditions where the genes of the heat-shock response are overexpressed. This finding is consistent with the proposed role of RNase G in regulating the *S. maltophilia* heat-shock response. Notably, expression of *emrA* and *emrB* is not increased upon heat-shock conditions, strongly suggesting they are not involved in the heat-shock-mediated quinolone resistance of *S. maltophilia*.

## Conclusion

Differing to other organisms in which high-level quinolone resistance is usually due to mutations at the genes encoding the bacterial topoisomerases, this type of quinolone-resistance mutations have never been described neither in *S. maltophilia* clinical isolates nor in the case of *in vitro* selected quinolone resistant mutants of this bacterial species (Ribera et al., 2002; Valdezate et al., 2002; Garcia-Leon et al., 2014b). While in some isolates quinolone resistance is associated to overexpression of multidrug eﬄux pumps (Alonso and Martinez, 2001; Sanchez et al., 2004; Garcia-Leon et al., 2015), some other mechanisms of resistance remain to be explored in this pathogen. Herein, we show that heat-shock induces a phenotype of quinolone resistance in *S. maltophilia*. In addition, RNase G regulates the expression of heat-shock responding genes, hence modulating the susceptibility to quinolones of *S. maltophilia*. Our work strongly supports that heat-shock response, a well conserved system in different bacterial species, triggers quinolone resistance (at least in *S. maltophilia*). In this way, heat-shock response proteins might be good targets for the development of new antimicrobials to be used together with quinolones.

Some works have shown that nearly 3% of the bacterial genome contributes to the characteristic phenotype of resistance of a given bacterial species. (Fajardo et al., 2008) Among those genes which inactivation modifies the susceptibility to antibiotics, several of them belong to basic categories of bacterial physiology (in principle not directly linked with antibiotics), including general metabolism, transport, and regulation among others. The finding that the lack of RNase G renders quinolone resistance, by triggering *S. maltophilia* the heat-shock response, fits with this situation and indicates that novel, non-classical mechanisms, might be involved in the acquisition of antibiotic resistance by this pathogen.

## Transcriptomic Data Accession Number

The results of the transcriptomic analysis described in this article were deposited in the SRA (Sequence Read Archive) database of NCBI (accession number SRR2128156).

## Conflict of Interest Statement

The authors declare that the research was conducted in the absence of any commercial or financial relationships that could be construed as a potential conflict of interest.

## Footnotes

1 http://www.macrogen.com

2 http://bioinfogp.cnb.csic.es/tools/FIESTA
