# Supplementary material for: The inactivation of RNase G reduces the Stenotrophomonas maltophilia susceptibility to quinolones by triggering the heat shock response
Source: Front Microbiol. 2015 Oct 19;6:1068. doi: 10.3389/fmicb.2015.01068 (PMC4609926; doi:10.3389/fmicb.2015.01068)
Supplement: Supplementary file 1 [file Table_1.DOCX]

**Table S1. Genes differentially expressed (more than two fold) in the RNase deffective mutant ALB001 as compared with the wild-type parental strain D457**

| **log_2_ (RPKM D457)** | **log_2_ (RPKM ALB001)** | **log_2_ (RPKM ALB001/RPKM D457)** | **Symbol** | **CDS product** |
| --- | --- | --- | --- | --- |
| -1,92 | 1,03 | **2,95** | SMD_2514 | flp pilus assembly protein TadD%2C contains TPR repeats |
| 0,13 | 2,67 | **2,54** | SMD_2429 | Aerotaxis sensor receptor protein |
| 3,39 | 5,66 | **2,27** | tRNA-Ser |  |
| 7,06 | 9,33 | **2,27** | tRNA-Gln |  |
| -0,05 | 2,06 | **2,1** | SMD_3831 | hypothetical protein |
| **10,74** | **12,72** | **1,98** | **groES** | **heat shock protein 60 family co-chaperone GroES** |
| 3 | 4,95 | **1,95** | tRNA-Ser |  |
| -1,65 | 0,3 | **1,95** | SMD_1538 | hypothetical protein |
| -0,17 | 1,78 | **1,95** | SMD_1569 | integrase regulator R |
| 1,43 | 3,39 | **1,95** | C4.5 |  |
| 1,31 | 3,27 | **1,95** | SMD_2218 | hypothetical protein |
| 4,97 | 6,87 | **1,9** | SMD_1358 | marR family transcriptional regulator |
| **11,48** | **13,26** | **1,78** | **groEL** | **heat shock protein 60 family chaperone GroEL** |
| 0,36 | 2,14 | **1,78** | SMD_3866 | ATP%2FGTP-binding protein |
| -0,23 | 1,4 | **1,63** | SMD_0959 | hypothetical protein |
| 8,35 | 9,99 | **1,63** | tRNA-Met |  |
| **6,31** | **7,93** | **1,62** | **emrA** | **tripartite multidrug resistance system membrane fusion protein** |
| 1,81 | 3,4 | **1,59** | SMD_0360 | hypothetical protein |
| -0,05 | 1,54 | **1,59** | trbJ | Conjugative transfer protein TrbJ |
| 6,86 | 8,44 | **1,58** | SMD_3130 | Serine protease MucD%2FAlgY associated with sigma factor RpoE |
| 3,23 | 4,79 | **1,56** | SMD_1697 | Pirin |
| 5,75 | 7,29 | **1,55** | SMD_1359 | tripartite multidrug resistance system outer membrane protein |
| 7,28 | 8,82 | **1,54** | htpG | chaperone protein HtpG |
| -0,14 | 1,4 | **1,54** | SMD_2508 | flp pilus assembly protein RcpC%2FCpaB |
| 7,11 | 8,56 | **1,45** | rpoE | RNA polymerase sigma factor RpoE |
| **6,59** | **7,99** | **1,4** | **emrB** | **tripartite multidrug resistance system inner membrane protein** |
| 0,42 | 1,79 | **1,37** | trbF | Conjugative transfer protein TrbF |
| 1,71 | 3,07 | **1,37** | SMD_1111 | hypothetical protein |
| 1,69 | 3,05 | **1,37** | SMD_1303 | hypothetical protein |
| 1,55 | 2,92 | **1,37** | SMD_1543 | hypothetical protein |
| 0,89 | 2,26 | **1,37** | SMD_1686 | ECF sigma factor |
| 0,41 | 1,78 | **1,37** | SMD_2200 | hypothetical protein |
| -0,31 | 1,06 | **1,37** | gspL | General secretion pathway protein L |
| 4,45 | 5,83 | **1,37** | SMD_2488 | oxidoreductase SMc00968 |
| -0,03 | 1,34 | **1,37** | SMD_2501 | hypothetical protein |
| 0,12 | 1,49 | **1,37** | X | phage-related tail protein |
| 2,01 | 3,37 | **1,37** | SMD_3292 | hypothetical protein |
| -0,54 | 0,83 | **1,36** | SMD_1548 | Plasmid-related protein |
| **9,48** | **10,8** | **1,32** | **dnaK** | **chaperone protein DnaK** |
| **6,91** | **8,19** | **1,27** | **hslV** | **ATP-dependent protease HslV** |
| 2,19 | 3,43 | **1,24** | SMD_0067 | hypothetical protein |
| 2,14 | 3,38 | **1,24** | SMD_1149 | Phosphoribosyl-dephospho-CoA transferase |
| 3,92 | 5,16 | **1,24** | nrdF | ribonucleotide reductase of class Ia aerobic%2Cbeta subunit |
| 4,89 | 6,1 | **1,21** | msrB | peptide methionine sulfoxide reductase MsrB |
| 2,78 | 3,97 | **1,2** | SMD_3299 | hypothetical protein |
| **7,11** | **8,29** | **1,18** | **clpB** | **clpB protein** |
| 6,15 | 7,32 | **1,17** | SMD_2129 | cointegrate resolution protein T |
| 0,31 | 1,48 | **1,17** | SMD_2214 | LmbE-like protein |
| 3,54 | 4,71 | **1,17** | SMD_3298 | hypothetical protein |
| 7,52 | 8,69 | **1,17** | hslU | ATP-dependent hsl protease ATP-binding subunit HslU |
| 1,26 | 2,43 | **1,17** | SMD_3750 | inner membrane protein YqjF |
| 3,41 | 4,55 | **1,14** | SMD_2443 | hypothetical protein |
| 4,24 | 5,39 | **1,14** | tRNA-Ser |  |
| 2,21 | 3,35 | **1,13** | SMD_1908 | major facilitator superfamily permease |
| 4,18 | 5,31 | **1,13** | SMD_4203 | marR family transcriptional regulator |
| 5,84 | 6,97 | **1,12** | glnA | glutamine synthetase |
| 8,26 | 9,37 | **1,11** | grpE | heat shock protein GrpE |
| 8,38 | 9,49 | **1,11** | dnaJ | chaperone protein DnaJ |
| 3,04 | 4,14 | **1,1** | C4.11 |  |
| 7,36 | 8,46 | **1,1** | tRNA-Lys |  |
| 1,33 | 2,43 | **1,1** | SMD_1572 | hypothetical protein |
| 2,45 | 3,55 | **1,1** | SMD_2931 | hypothetical protein |
| 6,7 | 7,78 | **1,08** | SMD_1751 | Zn-dependent hydrolases%2C including glyoxylases |
| 4,65 | 5,73 | **1,08** | SMD_2221 | hypothetical protein |
| 3,89 | 4,96 | **1,07** | SMD_2692 | N-formylglutamate deformylase |
| 7,35 | 8,43 | **1,07** | yybP-ykoY |  |
| 6,88 | 7,93 | **1,05** | SMD_3335 | hypothetical protein |
| -0,08 | 0,97 | **1,04** | SMD_1571 | hypothetical protein |
| 4,33 | 5,38 | **1,04** | SMD_2499 | merR family transcriptional regulator |
| 3,87 | 4,9 | **1,04** | SMD_3710 | G:T%2FU mismatch-specific uracil%2Fthymine DNA-glycosylase |
| 4,15 | 5,18 | **1,03** | SMD_2188 | TetR family transcriptional regulator |
| 9,51 | 10,52 | **1,01** | SMD_0137 | hypothetical protein |
| 7,09 | 6,08 | **-1,01** | SMD_1255 | DegT%2FDnrJ%2FEryC1%2FStrS aminotransferase |
| 1,46 | 0,45 | **-1,01** | SMD_1544 | Plasmid related protein |
| 6,81 | 5,8 | **-1,01** | flgC | flagellar basal-body rod protein FlgC |
| 5,87 | 4,86 | **-1,01** | SMD_2150 | superfamily I DNA%2FRNA helicase protein |
| 2,85 | 1,84 | **-1,01** | SMD_2195 | hypothetical protein |
| 5,11 | 4,1 | **-1,01** | SMD_2313 | macrolide-specific efflux protein MacA |
| 6,58 | 5,55 | **-1,02** | SMD_0040 | hypothetical protein |
| 7,33 | 6,31 | **-1,02** | SMD_0203 | hypothetical protein |
| 2,84 | 1,82 | **-1,02** | SMD_3985 | TonB-dependent receptor |
| 4,23 | 3,2 | **-1,03** | SMD_0894 | lipoprotein |
| 3,89 | 2,86 | **-1,03** | SMD_2149 | LuxR family two component transcriptional regulator |
| 5,88 | 4,84 | **-1,05** | SMD_0201 | cAMP-binding proteins-catabolite gene activator and regulatory subunit of cAMP-dependent protein kinases |
| 1,72 | 0,67 | **-1,05** | SMD_0905 | PE-PGRS FAMILY PROTEIN |
| 7,68 | 6,61 | **-1,06** | SMD_2072 | hypothetical protein |
| 7 | 5,95 | **-1,06** | oprP | phosphate-specific outer membrane porin OprP Pyrophosphate-specific outer membrane porin OprO |
| 3,78 | 2,7 | **-1,07** | kdpC | potassium-transporting ATPase subunit C |
| 5,93 | 4,86 | **-1,07** | pilJ | pilus biogenesis protein |
| 5,98 | 4,9 | **-1,08** | SMD_0202 | adenylate cyclase |
| 5,7 | 4,62 | **-1,08** | SMD_2317 | hypothetical protein |
| 4,98 | 3,89 | **-1,09** | tRNA-Leu |  |
| 3,01 | 1,92 | **-1,09** | tnpA2 |  |
| 2,77 | 1,68 | **-1,09** | copC2 | Copper resistance protein C precursor |
| 2,66 | 1,57 | **-1,09** | SMD_2667 | hypothetical protein |
| 2,53 | 1,44 | **-1,09** | SMD_3572 | Universal stress protein family |
| 3,38 | 2,28 | **-1,09** | SMD_3753 | Secreted and surface protein containing fasciclin-like repeats |
| 2,4 | 1,31 | **-1,09** | SMD_3967 | TonB-dependent receptor |
| 5,51 | 4,41 | **-1,1** | flgA | flagellar basal-body P-ring formation protein FlgA |
| 2,04 | 0,93 | **-1,12** | SMD_0895 | hypothetical protein |
| 7,32 | 6,2 | **-1,12** | smeZ | RND efflux system%2C inner membrane transporter CmeB |
| 5,81 | 4,69 | **-1,12** | SMD_3562 | hypothetical protein |
| 4,84 | 3,71 | **-1,13** | SMD_4054 | hypothetical protein |
| 10,04 | 8,89 | **-1,15** | SMD_0456 | hypothetical protein |
| 8,95 | 7,8 | **-1,15** | SMD_0457 | hypothetical protein |
| 4,19 | 3,04 | **-1,15** | SMD_2335 | hypothetical protein |
| 2,37 | 1,22 | **-1,15** | O | phage capsid scaffolding protein |
| 4,21 | 3,04 | **-1,17** | smeU2 | short-chain dehydrogenase%2Freductase |
| 3,92 | 2,74 | **-1,17** | SMD_2312 | macrolide export ATP-binding%2Fpermease MacB |
| 4,47 | 3,3 | **-1,18** | SMD_0250 | phage tail assembly protein |
| 4,73 | 3,54 | **-1,19** | SMD_0252 | phage tail fiber protein |
| 4,55 | 3,33 | **-1,22** | SMD_0246 | hypothetical protein |
| 2,25 | 1,03 | **-1,22** | msrA | peptide methionine sulfoxide reductase MsrA |
| 0,78 | -0,43 | **-1,22** | SMD_0890 | plasmid stabilization protein |
| 1,66 | 0,44 | **-1,22** | SMD_0956 | hypothetical protein |
| 0,83 | -0,39 | **-1,22** | SMD_1137 | signal peptide protein |
| 1,12 | -0,1 | **-1,22** | SMD_1612 | hypothetical protein |
| 0,68 | -0,54 | **-1,22** | SMD_2277 | hypothetical protein |
| 1,19 | -0,03 | **-1,22** | SMD_2279 | hypothetical protein |
| 1,37 | 0,15 | **-1,22** | SMD_2290 | RebB protein |
| 1,12 | -0,1 | **-1,22** | SMD_2363 | hypothetical protein |
| 1,94 | 0,72 | **-1,22** | SMD_2415 | hypothetical protein |
| 1,08 | -0,14 | **-1,22** | SMD_2526 | hypothetical protein |
| 2,22 | 1,01 | **-1,22** | SMD_2547 | MgtC%2FSapB transporter |
| 6,2 | 4,98 | **-1,22** | SMD_3160 | hypothetical protein |
| 0,97 | -0,25 | **-1,22** | W | phage baseplate assembly protein |
| 1,7 | 0,48 | **-1,22** | SMD_3609 | hypothetical protein |
| 5,57 | 4,36 | **-1,22** | SMD_3725 | methyl-accepting chemotaxis protein I serine chemoreceptor protein |
| 6,71 | 5,48 | **-1,24** | SMD_4079 | chemotaxis protein |
| 9,72 | 8,48 | **-1,25** | mtnX | 2-hydroxy-3-keto-5-methylthiopentenyl-1-phosphatephosphatase related protein |
| 7,61 | 6,35 | **-1,26** | SMD_3161 | methyl-accepting chemotaxis protein I serine chemoreceptor protein |
| 4,53 | 3,27 | **-1,27** | SMD_2421 | hypothetical protein |
| 6,37 | 5,1 | **-1,27** | SMD_3236 | chemotaxis response regulator containing a CheY-like receiver domain and a methylesterase domain |
| 4,38 | 3,1 | **-1,28** | SMD_2655 | hypothetical protein |
| 2,05 | 0,75 | **-1,3** | SMD_2294 | hypothetical protein |
| 5,67 | 4,35 | **-1,32** | tolA | TolA protein |
| 8,45 | 7,13 | **-1,32** | SMD_0614 | hypothetical protein |
| 3,93 | 2,61 | **-1,32** | SMD_1124 | general stress protein |
| 4,4 | 3,07 | **-1,32** | SMD_3671 | hypothetical protein |
| 0,92 | -0,45 | **-1,37** | B | phage capsid and scaffold |
| 10,26 | 8,87 | **-1,39** | pilA | type IV pilin PilA |
| 1,62 | 0,21 | **-1,41** | SMD_1607 | peptidase S14%2C ClpP |
| 4,58 | 3,15 | **-1,43** | SMD_0251 | phage tail assembly protein I |
| 4,87 | 3,44 | **-1,43** | SMD_0680 | hemin transport protein |
| 1,25 | -0,19 | **-1,44** | SMD_1549 | hypothetical protein |
| 3,08 | 1,64 | **-1,44** | SMD_2304 | hypothetical protein |
| 0,74 | -0,7 | **-1,44** | SMD_2308 | F420-dependent glucose-6-phosphate dehydrogenase |
| 0,92 | -0,52 | **-1,44** | SMD_2310 | Oxidoreductase |
| 3,32 | 1,88 | **-1,44** | SMD_2394 | hypothetical protein |
| 0,94 | -0,51 | **-1,44** | gspK | General secretion pathway protein K |
| 6,75 | 5,27 | **-1,48** | pstS | phosphate ABC transporter%2C periplasmic phosphate-binding protein PstS TC 3.A.1.7.1 |
| 3,99 | 2,5 | **-1,48** | SMD_1592 | hypothetical protein |
| 1,27 | -0,21 | **-1,48** | P | phage terminase%2C ATPase subunit |
| 4,7 | 3,2 | **-1,5** | SMD_3017 | phage protein |
| 3,99 | 2,48 | **-1,52** | phoU | phosphate transport system regulatory protein PhoU |
| 2,45 | 0,91 | **-1,54** | SMD_1603 | hypothetical protein |
| 5,94 | 4,35 | **-1,6** | SMD_1156 | Histone acetyltransferase HPA2 and related acetyltransferases |
| 4,62 | 2,99 | **-1,63** | SMD_0253 | hypothetical protein |
| 2,34 | 0,7 | **-1,63** | SMD_0955 | hypothetical protein |
| 1,62 | -0,01 | **-1,63** | SMD_1596 | hypothetical protein |
| 1,94 | 0,31 | **-1,63** | SMD_1597 | hypothetical protein |
| 2,06 | 0,43 | **-1,63** | SMD_1600 | hypothetical protein |
| 3,14 | 1,51 | **-1,63** | SMD_2245 | hypothetical protein |
| 1,8 | 0,17 | **-1,63** | SMD_2288 | RebB protein |
| 1,99 | 0,36 | **-1,63** | SMD_2291 | hypothetical protein |
| 1,48 | -0,15 | **-1,63** | gspI | General secretion pathway protein I |
| 1,59 | -0,04 | **-1,63** | SMD_2513 | type II secretion system protein |
| 5,44 | 3,73 | **-1,7** | SMD_0254 | hypothetical protein |
| 2,73 | 0,98 | **-1,75** | SMD_2515 | hypothetical protein |
| 1,83 | 0,03 | **-1,8** | SMD_2396 | Ferric siderophore transport system%2C periplasmic binding protein TonB |
| 4,28 | 2,45 | **-1,83** | SMD_3363 | hypothetical protein |
| 2,98 | 1,12 | **-1,86** | SMD_0433 | hypothetical protein |
| 2,92 | 1,04 | **-1,88** | SMD_2292 | hypothetical protein |
| 1,48 | -0,47 | **-1,95** | SMD_2278 | hypothetical protein |
| 2,48 | 0,53 | **-1,96** | SMD_0891 | hypothetical protein |
| 0,37 | -1,59 | **-1,96** | SMD_1520 | hypothetical protein |
| 0,41 | -1,55 | **-1,96** | copD2 | Copper resistance protein D |
| 2 | 0,05 | **-1,96** | smmB | hypothetical protein |
| 4,66 | 2,66 | **-2** | pstA | phosphate transport system permease protein PstA TC 3.A.1.7.1 |
| 2,42 | 0,36 | **-2,07** | SMD_0442 | major facilitator family transporter |
| 2,81 | 0,72 | **-2,09** | mgtC | Mg2 transport ATPase protein C |
| 0,69 | -1,53 | **-2,22** | SMD_1575 | chromosome partitioning ATPase in PFGI-1-like cluster%2C ParA-like |
| 1,26 | -0,96 | **-2,22** | SMD_2213 | type 12 methyltransferase |
| 2,4 | 0,18 | **-2,22** | SMD_2289 | RebB protein |
| 3,35 | 1,13 | **-2,22** | smmC | heavy metal RND efflux outer membrane protein%2CCzcC family |
| 5,59 | 3,32 | **-2,26** | pstC | phosphate transport system permease protein PstC TC 3.A.1.7.1 |
| 5,8 | 3,49 | **-2,31** | dctA | C4-dicarboxylate transport protein |
| 0,99 | -1,45 | **-2,44** | SMD_0111 |  |
| 0,86 | -1,58 | **-2,44** | SMD_1551 | periplasmic protein TonB%2C links inner and outer membranes |
| 2,29 | -0,15 | **-2,44** | SMD_1594 | hypothetical protein |
| 0,72 | -1,73 | **-2,44** | SMD_1687 | FecR protein |
| 2,17 | -0,27 | **-2,44** | SMD_3277 | hypothetical protein |
| 5,02 | 2,43 | **-2,59** | pstB | phosphate transport ATP-binding protein PstB TC 3.A.1.7.1 |
| 1,78 | -0,85 | **-2,63** | SMD_2506 | prepilin peptidase transmembrane protein |
| 5,23 | 2,34 | **-2,9** | SMD_2330 | hypothetical protein |
| 2,56 | -0,4 | **-2,95** | GpU | phage-related tail protein |
| 1,99 | -1,1 | **-3,09** | SMD_2559 | DSBA oxidoreductase |
| 2,34 | -0,75 | **-3,09** | FII | major tail tube protein |
| 4,82 | 1,51 | **-3,3** | mgtA | Mg2 transport ATPase%2C P-type |
| 5,81 | 1,62 | **-4,19** | SMD_3053 | hypothetical protein |
